# First-Principles Correlated Approach to the Normal State of Strontium Ruthenate

**DOI:** 10.1038/srep43033

**Published:** 2017-02-21

**Authors:** S. Acharya, M. S. Laad, Dibyendu Dey, T. Maitra, A. Taraphder

**Affiliations:** 1 Department of Physics, Indian Institute of Technology, Kharagpur, Kharagpur 721302, India; 2 Physics department, Kings College London, WC2R 2LS, UK; 3 Institute of Mathematical Sciences, Taramani, Chennai 600113, India; 4 Max-Planck Inst. fuer Physik Komplexer Systeme, 38 Noethnitzer Strasse, 01187 Dresden, Germany; 5 Department of Physics, Indian Institute of Technology, Roorkee, Roorkee 247667, India; 6 Centre for Theoretical Studies, Indian Institute of Technology Kharagpur, Kharagpur 721302, India

## Abstract

The interplay between multiple bands, sizable multi-band electronic correlations and strong spin-orbit coupling may conspire in selecting a rather unusual unconventional pairing symmetry in layered Sr_2_RuO_4_. This mandates a detailed revisit of the normal state and, in particular, the *T*-dependent incoherence-coherence crossover. Using a modern first-principles correlated view, we study this issue in the actual structure of Sr_2_RuO_4_ and present a unified and quantitative description of a range of unusual physical responses in the normal state. Armed with these, we propose that a new and important element, that of dominant multi-orbital charge fluctuations in a Hund’s metal, may be a primary pair glue for unconventional superconductivity. Thereby we establish a connection between the normal state responses and superconductivity in this system.

Unconventional superconductivity (USC) in layered Sr_2_RuO_4_ has long attracted intense attention^1^ owing to the expectation that it is a superconducting analogue of superfluid ^3^He, with a spin-triplet, odd-parity and chiral order parameter, described by Δ(*k*) ≃ (*k*_*x*_ + *ik*_*y*_)*z*^2^. Such a state would support half-quantum vortices and topologically protected chiral Majorana modes at the sample edges or on domain walls, which would be of fundamental interest. However, a surge of recent data points toward a much more interesting picture. A range of data also attests to the presence of line nodes in the SC gap function^3^. Moreover, presence of sizable spin-orbit coupling (SOC) and multi-orbital character of the system dictate that the multi-band pair function reflect spin-orbital entanglement; *i.e*., it cannot be classified as spin singlet or triplet^1^. It is fair to say that determination of the actual pair symmetry in Sr_2_RuO_4_ continues to be a fascinating open issue. Since the USC is an instability of the highly correlated Fermi liquid (FL) state in Sr_2_RuO_4_, resolution of this puzzle mandates a proper microscopic description of the normal state itself. Over the years, extensive experimental studies reveal (*i*) a *T*-dependent incoherence-coherence (IC-C) crossover from an incoherent high-*T* metal to a strongly correlated FL metal. The latter obtains only below *T*_*EL*_ ≃ 20–25 K, while signatures of an unusual metallic state^1^ are evidenced in a range of transport and magnetic fluctuation data for *T* > *T*_*FL*_. (*ii*) A careful de-Haas van-Alphen (dHvA)^4^ study has mapped out the low-*T*(<*T*_*FL*_) multi-sheeted Fermi surface (FS). Very recent correlated first-principles calculations^5^,^6^,^7^ show that it is necessary to adequately include the complex interplay between one-electron band structure, local multi-band interactions and SOC to get a quantitative accord with dHvA data. Moreover, the IC-C crossover has been interpreted in terms of Hund’s metal physics^8^,^9^,^10^, where sizable influence of Hund coupling (*J*_*H*_) drastically reduces the lattice-FL crossover scale. However, there is still no consensus on the details of the normal state (spin and charge) fluctuation spectra that can generate the specific pairing interaction needed to describe the SC pair symmetry constrained by multitude of data.

## Model and Formalism

Motivated thereby, we undertake a correlated first-principles theoretical approach using a combination of density functional theory and dynamic mean-field theory (DFT + DMFT) to address these issues. As an impurity solver, we use continuous-time quantum Monte Carlo (CTQMC)^11^, extended to low *T*. We use the Fermi surface data obtained from GGA + SO + DMFT that agree beautifully with the multi-sheeted Fermi surface from dHvA^4^ and calculate the single-particle Green’s function, self-energy, exponents, scaling features and scattering rates in detail in the temperature range 400 K to 12 K. Emergence of a possible low energy scale from these calculations points towards an IC-C crossover. The two-particle quantities, like spin and charge susceptibilties, and corresponding scaling features, provide a more accurate quantitative estimate of the crossover scale. We further address the transport and NMR responses for Sr_2_RuO_4_ over a similar temperature range and show their excellent accord with experimental results. We find, for the first time, that nearly singular inter-orbital charge fluctuations involving the *Ru*–4*d xy, yz, zx* bands, arise due to the complex interplay between orbital-selectivity and SOC. The implications of this on various proposals for the emergence of USC (from a correlated FL state in Sr_2_RuO_4_) are discussed.

**Figure 1:**
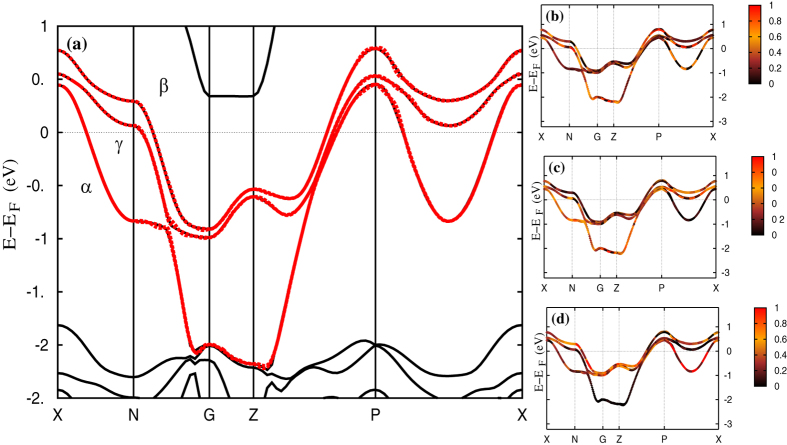
Left panel: Band structure (black solid lines) for *Sr*_2_*RuO*_4_ with SO coupling and Wannier fitting (red dotted lines) for the bands crossing the Fermi level. The bands with dominant *d*_*xz*_, *d*_*yz*_ and *d*_*xy*_ characters are shown as *α, β* and *γ* bands. Right panel: Band characterization of the Wannier fit bands for *Sr*_2_*RuO*_4_. The contribution of the *d*_*xy*_, *d*_*xz*_ and *d*_*yz*_ Wannier orbitals to the fitted bands are shown in (**b**–**d**) respectively through the color codes. Highest contribution is color red while black stands for the lowest. This intricate momentum-dependent interband mixing is a consequence of spin-orbital entanglement arising from sizable spin-orbit coupling (SOC) in Sr_2_RuO_4_.

Band structure calculations were performed in the real body-centered tetragonal (BCT-space group *I*4/*mmm*-139) structure. We first perform ab-initio density functional theory calculations within GGA and GGA + SO for *Sr*_2_*RuO*_4_ using the full potential linearized augmented plane-wave (FP-LAPW) method as implemented in the WIEN2k code^12^. We perform Wannierization of the Wien2k output bands around the Fermi level via interface programs like WANNIER90^13^, WIEN2WANNIER^14^. This in turn, gives us the Wannier orbital-projected bands around the Fermi level which serve as inputs of the DMFT self-consistency calculation. Three *t*_2*g*_ bands, comprising a two-dimensional *xy*-like *γ* band and a quasi-one-dimensional *β (xz*-*yz*-like) band, both electronlike, and a quasi-1D holelike *α*-band (*xz*-*yz*-like) cross *E*_*F*_. The bands and their Wannier fits are now shown along the symmetry directions in the reduced Brillouin zone (Fig. 1). It is clear that there is a sizable variation in the orbital character of the three *t*_2*g*_ states as one traverses the Fermi pockets in momentum space: this is a consequence of the intricate interplay between inter-band mixing, modified to reflect spin-orbital entanglement in the band structure due to strong spin-orbit coupling (SOC). In line with several data that point to sizable SOC, this aspect is also reflected in the fact that the FS topology is correctly reproduced in DFT only when SOC is included. However, sizable multi-band electronic correlations are mandatory to obtain quantitative accord with the dHvA FS^7^. We will see that the interplay between multiband correlations and SOC will also be crucial to quantitatively describe the IC-C crossover. Thus, use of a realistic Hubbard model for the *t*_2*g*_ bands with real structural input and SOC is mandatory.

Finally, we write down the three band *t*_2*g*_ Hubbard model


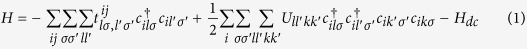


here, 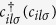 creates (destroys) an electron with spin *σ* in the Wannier state with orbital quantum number *l (l* = *xy, xz, yz*) at site i. *H*_*dc*_ is the double-counting correction. 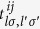 are the hopping integrals with SO coupling (*l* ≠ *l*′) and on-site energy matrix elements (*l* = *l*′). In the *D*_4*h*_ site symmetry, the *t*_2*g*_ states split into a *b*_2*g*_ singlet (*xy*) and *e*_*g*_ doublet (*xz, yz*), with *ε*_*xz*_ −*ε*_*xy*_ = *E*_*cf*_ ≃ 120 meV being the crystal field splitting. The terms *U*_*ll*′*kk*′_ are elements of the screened Coulomb interaction tensor. For *t*_2*g*_ states the terms are *U*_*ll*′*ll*′_ = *U*_*l,l*′_ = *U* − 2*Jδ*_*ll*′_ which is the direct screened Coulomb interaction. The exchange term is *U*_*ll*′*l*′*l*_ = *J*. Pair hopping is *U*_*lll*′*l*′_ = *J* and spin flip is *U*_*ll*′*l*′*l*_ = *J*. While the Hamiltonian formulation in our case is similar to what Zhang *et al*. used^7^, the only difference is the usage of isotropic *U*_*ll*_ = *U* in our case instead of the anisotropic on site intra-orbital interaction that Zhang *et al*. uses. We argue in the subsequent sections that the Wannier orbitals having different bandwidths *W*_*ll*_, the effective *U*_*ll*_/*W*_*ll*_ is different for different orbitals leading to self energies Σ_*ll*_ which is different for each Wannier orbitals, hence does not require choice of an orbital specific anisotropic *U*_*ll*_ at the first place.

## Results and Discussions

We begin by discussing our first-principles correlated approach including SOC, which gives the actual correlated Fermi surface for Sr_2_RuO_4_. The bare SOC (≃90–130 meV) is roughly of the order of the crystal field splitting at the DFT level. Inclusion of sizable *d*-shell correlations has many effects: (i) Correlation effects are larger for the *xy*-orbital states, since they lie lower in energy and are more populated. On the other hand, the smaller band widths of the *d*_*xz,yz*_ orbital states mean a larger effective *U*_*ll*_/*W*_*ll*_ (*l* = xz, yz) ratio for these. This would similarly mean that the degree of coherence is different in different orbitals, a measure of which can be the self energy for the individual orbitals. This essentially mimics the situation derived recently by Zhang *et al*. This is indeed the basic mechanism that sets the stage for orbital-selectivity to emerge. In the subsequent sections all the results we discuss are for *U* = 3.0*eV* and *J* = 0.4*eV*^10^ with *H*_*dc*_ = 7.21 eV. The self energies Σ(*iω*) are discussed for these correlation parameters and for a range of temperatures in later sections.

(ii) Due to inter-orbital charge transfer caused by the interaction terms associated with ∑_*i,σ,σ*′_*n*_*i,xy,σ*_*n*_*i,xz(yz*),*σ*′_, the bare crystal field splitting is renormalized already at the Hartree level, to an effective value less than its bare DFT value. Since the SOC coupling constant is weakly enhanced by correlations (a simple way to see this is that the static Hartree-Fock contribution from the Hund term directly renormalizes the bare SOC, so 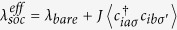), the ratio of the effective SOC to the effective crystal field splitting is enhanced upon switching on electronic correlations. This means that it is no longer possible to disentangle orbital and spin degrees of freedom, a feature which must have far-reaching consequences for the detailed symmetry of the pair wavefunction in Sr_2_RuO_4_.

**Figure 2:**
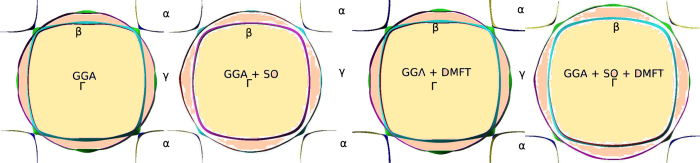
Fermi surfaces for Sr_2_RuO_4_ calculated using GGA, GGA + SO, GGA + DMFT, GGA + SO + DMFT. In consistency with the band structure we show the *α, β* and *γ* Fermi sheets and the high symmetry Γ point. In agreement with Zhang *et al*., We find that SOC is essential to derive the quantitatively correct Fermi sheets as seen in dHvA experiment of Bergemann *et al*.

Both these effects directly bear upon the renormalized electronic structure and the Fermi surface topology. In Fig. 2, we exhibit the GGA, GGA + DMFT, GGA + SOC and GGA + SOC + DMFT Fermi surfaces. It is clear that GGA + DMFT alone gives Fermi surfaces in discord with dHvA data, and that inclusion of SOC is mandatory to obtain correct Fermi surface sheets with regard to their shapes and size^7^.

In addition, these changes in the bare DFT parameters cause changes in dynamical spectral weight transfer, caused by higher order terms in the self-energy in DMFT. Thus, effective mass enhancements will be orbital-dependent in the low-*T* FL state, as is known^4^, and, given different effective *U*/*W* for each band, different bands are narrowed down to differing extent. The average effective mass enhancement at 30 K is *O*(3–4), in excellent accord with both dHvA estimates and specific heat data. The coherent part of the “renormalized band structure” could be tested against ARPES band structures in future. More importantly, we find that the orbital character of the three *t*_2*g*_ bands is sizably k-dependent due to momentum-dependent spin-orbital entanglement. This is a crucial input when one considers the construction of a “pair interaction” to obtain USC: the pair interaction must also reflect this spin-orbital entanglement. We leave this aspect for future studies.

In the subsequent sections we discuss (i) the single- and two-particle responses in the normal phase of *Sr*_2_*RuO*_4_, (ii) the NMR and transport to make a case for the (a) IC-C crossover in *Sr*_2_*RuO*_4_ and (b) the possible form factor for pairing fluctuation for the unconventional superconductivity below 1.5 K. Extensive scaling analyses and fitting of the single- and two-particle data are performed in the following section. Such analyses are very useful to charaterize the NFL and FL responses^15^ of the system. It is primarily the values of these parameters that underline the quantum crossover.

### Normal State Responses

#### Single-particle Quantities

**Figure 3:**
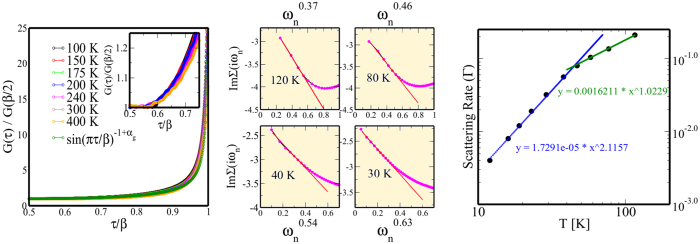
Imaginary parts of the scaled one-electron Green functions (left) as function of *τ*/*β*. For *T* > *T*_*FL*_ ≃ 30 K, it exhibits a clear local quantum-critical scaling behavior, implying strange metallicity in Sr_2_RuO_4_ above 30 K. The low-energy features for *G(τ*)/*G(β*/2) is zoomed in (inset) to show the near perfect scaling collapse at and around low energies (*τ* = *β*/2). Within our numerical accuracies, the low energy scaling is valid for the whole range between 30 K to 400 K. The imaginary parts of the self-energy −*Im*Σ(*iω*_*n*_) (middle) as a function of *ω*_*n*_ shows clear anomalous power-law behavior up to high energy *O*(0.5) eV which further testifies to anomalous metallicity for *T* > 30 K. As the temperature decreases the exponent increases steadily towards the FL limit (linear in *ω*_*n*_). The scattering rate (Γ) that we get from the −ImΣ(*ω* = 0) shows clear crossover in the temperature dependence of the Γ from high temperature linear in *T* to low temperature *T*^2^ across ~30 K. This clearly shows the emergence of coherence at the single particle sector as well.

We now study the normal state responses in Sr_2_RuO_4_ in detail. In Fig. 3, we show the full one-electron local Green’s function in imaginary time, *G(τ*) as a function of *T*(>*T*_*FL*_). Very good collapse of all curves on to a single curve is clear and, upon careful fitting with a “local” quantum-critical form, 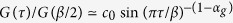 with *α*_*g*_ = 0.32. The inset zooms into the low-energy features for the *G(τ*)/*G(β*/2) (around *τ* = *β*/2) and shows that the low energy scaling is valid for the whole range between 30 K to 400 K. However, this conformal-invariant form is that expected for a locally quantum-critical metal, where the infra-red pole structure of *G(ω*) in a FL metal is supplanted by an infra-red branch-cut continuum form with anomalous fractional exponents. We fit the imaginary part of the self energy with the form −*Im*Σ(*ω*_*n*_) = *C* + *A(ω*_*n*_)^*α *^15 over the low energy Matsubara points. We extract the constant *C* and the exponent *α* therefrom. The evolution of *α* as a function of temperature shows that *Sr*_2_*RuO*_4_ is a bad metal for *T* > 30 K, and with lowering of temperatures, *α* increases (to 0.63 at *T* = 30 K) and possibly approaches the FL limit 1.0 at lower temperatures. Correspondingly, Fig. 3 also shows that this anomalous behavior of self-energies persists up to rather high energies. From the intercept *C* (−ImΣ(*ω* = 0)) we can also extract the scattering rate (Γ). The temperature dependence of Γ changes from high temperature linear in *T* to low temperature *T*^2^ across ~30 K. This clearly shows the emergence of LFL like coherence at the single particle sector as well. However, since it’s a crossover, it’s difficult to identify a clear boundary from these single particle features. At the next level we study the two-particle dynamic responses to further pinpoint the crossover temperature.

#### Two-particle Quantities

**Figure 4:**
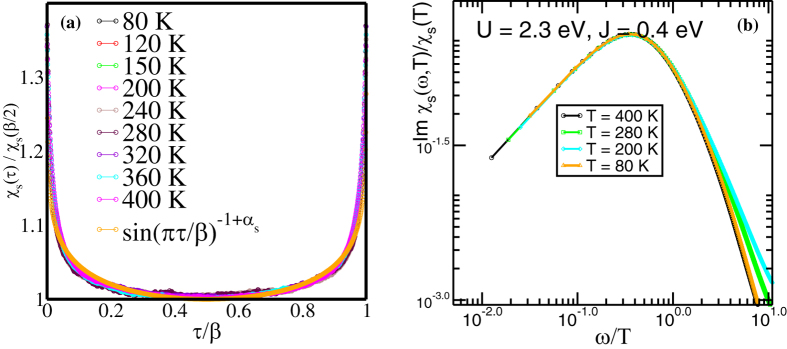
(**a**) Clear thermal collapse of *χ*_*s*_(*τ*) to a universal scaling function, given by 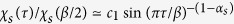, with *α*_*s*_ ≃ 0.93. (**b**) Analytically continued Im*χ*_*s*_(*ω, T*) with proper thermal scaling collapse for Im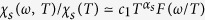 upto *ω*/*T* ~ 1.0 in the bad metallic phase above *T* ≃ 30 K.

**Figure 5:**
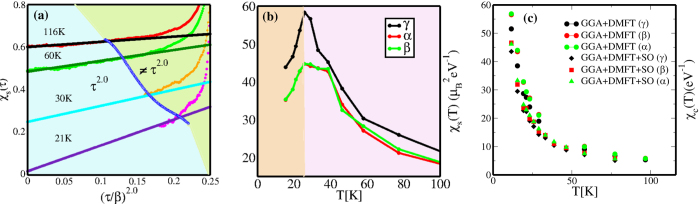
(**a**) Gradual restoration of correlated Landau Fermi liquidity as *T* is lowered below 30 K, as seen by the fact that the intercept on the *χ*_*s*_(*τ*) axis vanishes at ≃21 K. The *χ*_*s*_(*τ*) is *τ*^2^ (Fermi Liquid like) for finite low energies and finally deviate from *τ*^2^ across the blue boundary where incoherence sets in. With lower temperatures the FL liquid behavior sustains over larger energy ranges, showing predominance of coherence. (**b**) The incoherence-coherence crossover is reflected in the change in the *T*-dependent band-resolved spin susceptibilities around 25 K. (**c**) We show that the orbital-resolved charge susceptibilities are enhanced relative to the spin susceptibilities at low *T*, making them attractive candidates for an electronic pair glue. We also show that the magnitudes of the intra-orbital static charge susceptibilities *χ*_*c*_(*T*) decrease once *SO* coupling is included. However, both with and without *SO* coupling *χ*_*c*_(*T*) remain singular down to 12 K.

Interestingly, the dynamical spin susceptibility also exhibits similar scaling behavior: in Fig. 4(a), clear collapse of *χ*_*s*_(*τ*) to a universal scaling function, given by 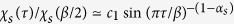, with *α*_*s*_ ≃ 0.93 is seen. This is precisely the form^16^,^17^,^18^ expected for an intermediate non-Landau Fermi liquid metallic *T*(>*T*_*FL*_) state, in a regime where one-electron coherence has not yet been achieved (it occurs at low *T* < *T*_*FL*_ in our DMFT, see below). Finally, we analytically continue the *χ*_*s*_(*τ*) data using maximum entropy method (MEM)^19^ and find a proper thermal scaling collapse for Im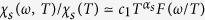 (Fig. 4(b)) upto *ω*/*T* ~ 1.0 in the bad metallic phase above *T* ≃ 30 K. We show the scaling collapse within the temperature range between 400 K to 80 K. However, the unscaled local susceptibilities *χ*_*s*_(*τ*) in Fig. 5(a) show something remarkable when *τ* is scaled as (*τ*/*β*)^2.0^. We clearly show the energy scales at each temperature across which the *χ*_*s*_(*τ*) deviates from (*τ*/*β*)^2.0^. Figure 5(a) shows that with lowering temperatures the energy scale over which *χ*_*s*_(*τ*) ~ (*τ*/*β*)^2.0^ increases, and, more importantly that the *intercept* approaches zero for *T* ≤ *T*_*FL*_ but remains finite for *T* > *T*_*FL*_, clearly showing up the emergence of FL coherence at low energies below *T*_*FL*_ ≃ 23 K. The static local susceptibilities as functions of temperature are obtained by integrating *χ*_*s*_(*τ, T*) over *τ*. In this way, the intra-orbital spin and charge susceptibilities^20^ are extracted over a range of temperature between 116 K and 12 K. The local irreducible vertex corrected intra-orbital static spin susceptibilities *χ*_*s*_(*T*) (Fig. 5(b)) show predominant Curie-Weiss nature at higher temperatures and the Curie-Weiss singularity gets cut-off around ~25 K showing emergence of low energy FL coherence. While this happens for all three orbitals (*α, β, γ*) the magnitude of the local static spin susceptibility for *γ* orbital, which has dominant contribution from *d*_*xy*_, is larger than rest of the two. Unlike spin-susceptibilities, Fig. 5(c) shows intra-orbital charge susceptibilities *χ*_*c*_(*T*) remain singular down to 12 K. This is the temperature upto which our CT-QMC works without numerical fluctuations and the data is mostly noise-free. However, due to computational constraints imposed by CT-QMC, we cannot go to temperatures lower than 12 K. In principle, the charge susceptibility should also saturate, but it may occur at a temperature lower than 12 K, but still higher than *T*_*c*_ for superconductivity. This limitation is common to all CTQMC solvers, and 12 K is the best that we could attain presently. Unlike spin susceptibilities, the intra-orbital charge susceptibilities for *α,β* orbitals have higher magnitudes than *γ* orbital. At this point, it is equally interesting to note that the intra-orbital *χ*_*c*_(*T*) does change in magnitude once the *SO* coupling is included. It seems with *SO* coupling the magnitude of the intra-orbital *χ*_*c*_(*T*) decreases, although they still remain singular. The behavior of *χ*_*s*_(*T*) and *χ*_*c*_(*T*) hint that although spin gets quenched below 25 K, charge fluctuations act as the soft mode in *Sr*_2_*RuO*_4_.

Microscopically, a reason for these findings is that the Hubbard *U* = 2.3 eV is larger than the band-width of xz, yz bands (*W*_*xz,yz*_ = 1.4 eV), while it is comparable to that of the *xy*-band. Thus, depending upon the orbital, one is effectively either in the intermediate (*xy*) or the strong-coupling (*xz, yz*) of the three-band Hubbard model, with non-integer occupation of each orbital. In the *xz, yz*-sector, we now expect a tendency to have orbital-selective Mott-like states. At high *T*, the inter-orbital hybridization is effectively rendered irrelevant by sizable *J*_*H*_ (Hund’s metal scenario), and thus one deals with an effective situation where there is strong scattering between metallic (*xy*) and effectively localized (*xz, yz*) carriers. In the local impurity model of DMFT, this implies that the latter cannot recoil during an interband scattering process, leading to emergence of recoil-less X-ray edge physics. The high-*T* infra-red singularities in one- and two-particle propagators we find in DMFT are thus associated with local processes akin to those occurring in the seminal orthogonality catastrophe^21^.

### NMR and Transport

These results offer direct insight into the high-*T* anomalies characteristic of *Sr*_2_*RuO*_4_. First, transport can now be rationalized solely in terms of the structure of *G*_*loc*_(*ω*) or Σ_*loc*_(*ω*), since local irreducible vertex corrections are negligible^22^ in multi-orbital DFT + DMFT. Using results above, we find that the *dc* resistivity, 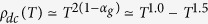, which is qualitatively consistent with observations above *T*_*FL*_ ≃ 30 K^1^ right up to 900 K. Correspondingly, the optical conductivity, 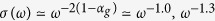. This also implies anomalous energy-dependent scattering rate, *τ*^−1^(*ω*) ≃ *ω*^1.0^, *ω*^1.3^, and energy-dependent effective mass enhancement, m*(*ω*)/*m*_*b*_ ≃ *ω*^*−*1.0^, *ω*^*−*1.3^. Remarkably, both these features seem to be in accord with data (see Fig. (4) of Katsufuji *et al*.^23^, and our results suggest a re-interpretation of the *T* > *T*_*FL*_ transport data within an incoherent metal scenario (indeed, no FL contribution is seen down to 0.03 eV in optical data for *T* > *T*_*FL*_). Moreover, the local critical form of the dynamical spin susceptibility permits rationalization of the anomalous neutron and NMR results above *T*_*FL*_ as follows: *y* = *ω*/*T*-scaling in *χ*_*s*_(*ω, T*) with an exponent (1 − *α*_*s*_) ≃ 0.93 implies that *ω*^0.933^Im*χ*_*s*_(*ω, T*) must be a universal scaling function of *y* for *T* > *T*_*FL*_. This is fully borne out by neutron scattering data above 30 K^24^, which is precisely the crossover scale for FL behavior. In addition, *α*_*s*_ = 0.933 is also close to the exponent of 1.0 used to fit the neutron scattering intensity for *ω* > 2.0 meV^26^,^25^. Finally, the full-width at half-maximum, related to the damping of magnetic excitations must scale as 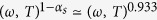, again in excellent accord with data. The NMR spin relaxation rate should vary as 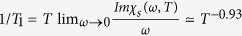, implying a sizably *T*-dependent (but increasing with reduction of *T*) 1/*T*_1_ for *T* > *T*_*FL*_: this is also not inconsistent with data^27^ for H||*c*. Thus, good accord with a range of normal state responses lends strong support to an intermediate-*T* local non-FL “strange” metallic state in Sr_2_RuO_4_.

Such non-FL “strange” metallic state is famous in cuprates. However, in contrast to high-T_*c*_ cuprates *Sr*_2_*RuO*_4_ shows a smooth crossover from the high-*T* non-FL metal to a correlated FL metal below *T* ≃ 30 K. Understanding the nature of this crossover is mandatory to uncover the microscopics of USC setting in below *T*_*c*_ = 1.5 K. We operate CT-QMC + DMFT down to *T* ≃ *O*(12) K which enables us to study this IC-C crossover in detail. This is also borne out by the observation that the orbital-dependent effective masses acquire sizable enhancement, become almost *T*-independent, along with drastically reduced scattering rate below *T* ≃ 30 K, in excellent accord with indications for a low-*T* correlated FL metal from optical data^23^. In fact, the effective masses for all orbitals are enhanced by a factor of about 3–4 at low *T*, in nice accord with specific heat data^25^. Since the out-of-plane resistivity also acquires a *T*^2^ dependence below *T*_*FL*_, it is likely that a dimensional crossover is implied by the non-FL-to-FL crossover. In a quasi-2D correlated FL, this crossover should occur when *k*_*B*_ ≃ *t*_⊥_, the one-electron hopping between weakly coupled layers. One might then wonder whether (and how) the strong incoherent metal signatures found for *T* > *T*_*FL*_ influence this crossover. We now provide a detailed analysis of this, showing how “high-*T*” incoherence indeed dramatically affects details of the low *T* FL state in ways incompatible with a simple one-electron band-structure view.

### The Incoherence-Coherence (IC-C) Crossover

We now present a physical argument that attempts to clarify the above results and those that follow, when the system crosses over to a Landau Fermi liquid (LFL) at low *T*. We are motivated in this by a similar work for quasi-1D organics^22^. The reason we can use this argument is that the *xz(α*), *yz(β*) band states are one-dimensional to a very good approximation at “high”-*T* > 100 K, and full three-dimensional coherence accompanied by restoration of correlated LFL metallicity only occurs below 25 K. Also, an additional point is that the interlayer hopping along *k*_*z*_ in Sr_2_RuO_4_ involves inter-orbital one-electron mixing between the *xz, yz* orbitals, since one-electron overlap between *xy(γ*)-orbital states is negligible in the BCT geometry. Physically, due to this interlayer mixing between *xz, yz* states at low *T*, the interband hybridization eventually gets relevant at a scale pushed dramatically downward by Hund metallicity, introducing recoil into the local impurity model above, and cuts off the infra-red singularities found for *T* > *T*_*FL*_. To consider this crossover as a function of *T*, we observe that: (*i*) the interlayer hopping, *t*_⊥_ ≃ 0.02 *eV*(200 *K*) ≪ *t*_*aσ,bσ*′_^1^, even in the renormalized DMFT electronic band structure, and (*ii*) at high *T*, the *c*-axis resistivity shows insulator-like behavior in contrast to the bad-metallic in-plane resistivity. These findings lend substance to our argument that a dimensional crossover from effectively decoupled 2*D* layers (at high *T*) to an anisotropic 3*D* state is involved in the IC-C crossover.

A description of the effect of *t*_⊥_ requires consideration of a model with coupled *RuO*_2_ layers. Since the interlayer hopping for the *d*_*xy*_ band is much smaller than for the *d*_*yz,zx*_ bands in the undistorted BCT structure, we are led to consider the effective model of two coupled quasi-1D xz, yz bands to facilitate the description of the IC-C crossover as a dimensional crossover, driven by increasing relevance of coherent one-electron inter-orbital mixing at lower *T*:


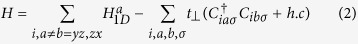


where 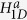 represents the Hamiltonian for the two quasi-1*D* bands (*a, b* denoting *xz, yz* bands), and the second term describes the intra- and inter-orbital overlap between the two bands. We now discuss the IC-C crossover and the associated dimensional crossover with a somewhat subtle analytic framework. For a system of coupled 1*D*-like chains as above, a description of this crossover by perturbation theory in *t*_⊥_ in the non-FL metal is beset with difficulties, and is valid only in the non-FL regime (which we find as above for *T* > *T*_*FL*_), but fails to reproduce the FL regime. Perturbation approaches in interaction, beginning from the free band structure work in the FL regime, but fail in the non-FL regime. An attractive way out is provided by a close generalization of a non-trivial argument developed in the context of coupled Luttinger chains^22^. In our case, though each RuO_2_ layer is connected via *t*_⊥_ to *z*_⊥_ nearest neighbors, with *z*_⊥_ → ∞, 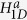 above does *not* represent two coupled Luttinger liquids, since both are coupled to the 2*D xy* band states via *U*′, *J*_*H*_ and sizable SOC.

Within this analytic framework, one needs a numerical solution for the full local self-energy, Σ_*a*_(*ω*), as an input, which we take from our DFT + DMFT calculation. Using this input, we can draw qualitative conclusions regarding the effect of non-FL metallicity at high-*T* on the non-FL to FL crossover at lower *T* as follows. In the non-FL regime, for each of the *xz, yz* bands, the in-plane self-energy 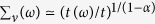, as found above. It is clear that *t*_⊥_ becomes relevant, inducing the dimensional crossover when 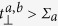, yielding the crossover scale 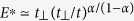. With *α* = 0.32 in our case, this yields E* ≃ 40 K, in good accord with the numerical estimate of 25 K in numerics.

Once *T* < E^*^, one ends up with an anisotropic correlated FL metal. In particular, when *t* ≪ *t* and at low energies, all one-particle quantities obey the scaling *ω*′ = *ω*/E^*^, and *T*′ = *T*/E^*^; i.e., *t*Σ(*ω, T*) = E^*^t_⊥_Σ′(*ω*′, *T*′) and *tG(ω, T*) = (E^*^/*t*_⊥_)*G*′(*ω*′, *T*′) where Σ and *G* are universal functions associated with the crossover. A low-frequency expansion of Σ in the FL regime gives the quasiparticle residue *Z* ≃ (*t*_⊥_/*t*)^*α*/(1−*α*)^ = E*/*t*_⊥_. The inter-layer resistivity, *ρ*_⊥_(*T*)/*ρ*_0_ = (*t*/E*)*R(T*/E*) with *R(x* ≪ 1) ∝ *x*^2^ and *R(x* ≫ 1) ∝ *x*^1−2*α*^. And the resistivity enhancement, *ρ*_⊥_(*T*)/*ρ*_0_ = *A(T*/*t*)^2^ with *A* = (*t*/*t*_⊥_)^3/(1−*α*)^. The resulting anisotropy of the Woods-Saxon ratio, *A*_*c*_/*A*_*ab*_ = (*a*/*c*)^2^*A* ≃ 1000 for *α* = 0.32, which is indeed in the right range^1^. Finally, the *c*-axis optical response is incoherent above E*, with a coherent feature carrying a relative weight ≃*Z*^2^ appearing at low-*T*, again in qualitative agreement with observations^23^. An obvious inference from the above is that increasing *T* should lead to a disappearance of the quasicoherent features in photoemission. This may already have been observed experimentally^28^. Thus, the IC-C crossover in Sr_2_RuO_4_ involves a multi-orbital based picture, where increasing relevance of the *c*-axis hopping between the quasi-1*D xz, yz* states drives restoration of LFL coherence (see below). At low-*T*, in the correlated FL regime, our LDA + SOC + DMFT(CTQMC) results also provide a consistent description of electronic correlations and lead to very good quantitative accord with dHvA and mass enhancements, as shown above. In our picture, therefore, the onset of LFL metallicity also arises from renormalization effects caused by local multiband electronic correlations (DMFT) in an orbitally anisotropic electronic structure characteristic of layered Sr_2_RuO_4_.

In the qualitative description of the non-FL to FL crossover above, the “high”-*T* non-FL metal is taken to be the local critical metal. The main advantage of the above analysis is that it gives a picture for this crossover (which is thus a multi-band dimensional crossover) with increasing relevance of the inter-layer one-electron hopping involving *xz, yz* states at lower *T*; since the SOC seems to become relevant at lower *T* reflected in the in-plane versus out-of-plane spin susceptibility anisotropy^1^. Related ideas have been advocated earlier^25^, but never demonstrated within first-principles correlated calculations. We re-emphasize that in our work, the non-LFL metal is *not* linked to 1*D* Luttinger liquid physics, but arises from incoherent metallicity, itself driven by a combination of orbital selectivity and Hund’s metallicity.

### Scenario for Electronic Glue

To further characterize this crossover and search for a dominant electronic pair “glue”, we computed the *T*-dependent local intra-orbital spin (*χ*_*s*_(*T*)) and charge susceptibilities (*χ*_*c*_(*T*) for each of *xy(γ*), *yz(β*), *xz(α*))(Fig. 5(c)) orbitals. In Fig. 5(b), we show *χ*_*s*_(*T*) from high- up to low *T*. Clear Curie-Weiss like behavior at high *T* smoothly crossing over to a high but relatively *T*-independent value at low *T* < 25 K is a manifestation of the non-FL-to-FL crossover. Moreover, we find a high-spin state (*S* = 1) on Ru, and thus a Hund metal, arising from sizable Hund coupling. Several points are in order: (*i*) details of the *T*-dependence of *χ*_*s*_(*T*) are in good accord with data if one assumes^26^ a “relaxor” form 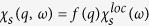 with 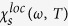 (Fig. 4(b)) evaluated by analytically continuing the Matsubara data. Due to enhancements at incommensurate *q*_*in*_ coming from the quasi-1D xz, yz bands (which enter via the factor *f(q*) in an RPA like view), the relevant quantity to compare is *χ*_*s*_(*q*_*in*_, *ω, T*). Assuming, in spirit of DMFT, that the *T*-dependence is dominantly in 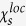, we find surprisingly good accord with data over the whole *T* range, right down to 25 K, where *χ*_*s*_(*q*_*in*_, *T*) flattens out. However, the orbital-resolved charge susceptibilities spring a surprise: as we pointed out before in Fig. 5, we show that *χ*_*c*_(*T*) for *xz, yz* is enhanced relative to *χ*_*xy,c*_(*T*), and, more importantly, that *χ*_*c*_(*T*) always increases at lower *T* without flattening out, at least down to 12 K (note that both *χ*_*s*_, *χ*_*c*_ are presented in real units). We expect *χ*_*c*_ to eventually approach an enhanced *T*-independent value at low *T* (since the metallic state immediately above *T*_*c*_ = 1.5 K is a LFL), but we are unable to access this possibility numerically below 12 K because of computational restrictions imposed by the CTQMC solver. It is also noteworthy that *χ*_*xy,c*_ is comparably enhanced at low *T*. Finally, we have performed CTQMC without and with SOC to analyze its role in generating enhanced charge fluctuations. We find (not shown) that the main qualitative trend remains unchanged and, in fact, *χ*_*c*_(*T*) is even larger without SOC. We observe that this slight *reduction* is caused by the fact that the SOC constant, *λ*, is *negative* in the *t*_2*g*_ shell in the *d*^4^ configuration relevant to Sr_2_RuO_4_.

Our finding implies that intra-band charge fluctuations arising from the quasi-1D *α, β* FS sheets (xz, yz orbital character) are the “softest”, though only marginally enhanced compared to *χ*_*xy,c*_(*T*). (the incommensurate spin susceptibility “flattens” out to sizably lower values at low *T*). Interestingly, the charge fluctuations in the xy-band closely track them, strongly suggesting a novel possibility for the SC mechanism, were these to be involved in the SC pair glue (see below). It is crucial to note that clear orbital “selectivity” reflected in our results is a consequence of the dynamical interplay between (DFT) structure (in particular, the crystal field splitting between the *xz, yz* and *xy* orbitals in the *D*_4*h*_ structure *and* a combination of *D* = 2(*xy*) and quasi-1*D(xz, yz*) band states), sizable multi-orbital correlations and spin-orbital entanglement due to SOC enhanced by correlations. Thus, our analysis bares a new, hitherto unappreciated factor: the “normal” state above *T*_*c*_ maybe close to being electronically “soft” in the inter-orbital charge channel. Very good accord with a wide range of normal state responses (dHvA Fermi surfaces, inelastic neutron scattering lineshapes and NMR, and incoherent metallicity of the “strange metal” type) we describe above lends strong credence to the feasibility of this conclusion.

We describe qualitatively the novel implications of these findings for the mechanism for USC in Sr_2_RuO_4_. We do not aim to provide a microscopic mechanism for USC in this system, but discuss how our results lead to a novel choice for the “pair glue”. The nearly soft intra-band charge fluctuation modes found above must overwhelm the incommensurate spin fluctuations at low *T* (just above *T*_*c*_), raising a hitherto neglected possibility that these might be primary sources for the “pair glue” leading to USC. If this be the case, our results provide support to some of the other mechanism(s)^29^,^30^,^31^.

Further, strong SOC also implies that these multi-orbital charge fluctuations are intrinsically entangled^6^ with mixed singlet-triplet magnetic fluctuations. Raghu *et al*.^29^ claim that USC primarily arises from the quasi-1D *xz, yz* bands. We emphasize that these authors already point out that intra-band and inter-band *charge* fluctuations could be involved in the pairing and in Sr_2_RuO_4_ strong SOC would nevertheless involve spin fluctuations as well. The interband proximity effect induces a secondary SC gap in the *xy* band^32^,^33^. Scaffidi *et al*.^30^ and Wang *et al*.^31^ claim, within a weak-coupling RG procedure, that all three *d*-bands are involved in SC. However, our results, along with earlier local density approximation (LDA) + DMFT ones^7^,^9^, show that Sr_2_RuO_4_ is more aptly characterized as a sizable-to-strongly correlated system. The RG procedures hitherto used for study of pair symmetry are valid in the weak coupling (*U* ≪ *W*) limit, but would be inadequate when *U* ≃ *W*, as appropriate here. Strictly speaking, it is possible that the weak coupling calculations identify the correct pair-symmetry if one could invoke analytic continuity from weak to the intermediate-coupling state: this maybe possible for Sr_2_RuO_4_, since the low-*T* state immediately above *T*_*c*_ is a correlated FL, which is analytically continuable in the Landau sense from a weakly correlated metal. At intermediate to strong coupling, however, a more suitable theoretical framework to study instabilities to ordered states should involve consideration of singularities in the two-particle vertices in competing particle-hole and particle-particle channels, for instance within a parquet approach. Such a study, however, is demanding, especially within first-principles correlated approaches, and is out of scope of our present work.

The emergent low-energy picture from our normal-state results is the following: already at high *T*, the Hund coupling results in *S* = 1 on each Ru site, which also controls the very low *T*_*FL*_ ≃ 25 K via a combination of Hund metal physics^10^ and a dimensional crossover (see above). At high *T*, both the SOC as well as the small (*t*_⊥_ ~ 0.1*t*_*aσ,bσ*′_) interlayer hopping are irrelevant, and one can view the system in terms of an orbitally degenerate quasi-1D (*xz*-*yz*) system coupled to a wider 2D *xy*-band via two-body Coulomb interactions in the *t*_2*g*_-sector. At low *T *< *T*_*FL*_, both (especially *t*_⊥_) become relevant, inducing both, spin-orbital entanglement and a dimensional crossover, resulting in an anisotropic quasi-3D correlated FL. Importantly, since sizable local interactions and SOC have already drastically renormalized the quasi-coherent part of the bare GGA band structure as shown in Fig. 1, it now follows that the “effective interactions” which finally cause cooper pairing should involve renormalized interactions. We restrict ourselves to qualitative but symmetry-based reasoning as the full treatment is beyond the scope of present work. With the assumption that SC primarily arises from the quasi-1D xz-yz bands (suggested by the maximally enhanced intra-band charge susceptibilities in the *xz, yz* sector in the normal-state DFT + DMFT calculation) and noticing that only these orbitals have non-negligible overlap with out-of-plane *t*_2*g*_ orbitals in the BCT structure, we are led to a picture similar to that proposed by Hasegawa *et al*.^34^. In the BCT structure, symmetry arguments lead one to a two-fold degenerate, multi-band spin-triplet and odd-parity pair state of the form


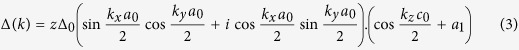


which reduces to the form Δ(*k*) = *z*Δ_0_(*k*_*x*_ + *ik*_*y*_)(*cos(k*_*z*_*c*_0_) + *a*_1_) for small *k*_*x*_, *k*_*y*_, where the *a*_1_ component can exist on symmetry grounds in Sr_2_RuO_4_^34^ (indeed, it results from the interband proximity-induced (*k*_*x*_ + *ik*_*y*_) gap on the *xy*-band). The form factor cos(*k*_*z*_*c*) immediately leads to horizontal line nodes at *k*_*z*_ = *π*/2*c* ± *δk*_0_ as long as |*a*_1_| < 1, as required from various data. The crucial point is that this choice naturally results from an effective interaction which primarily couples the *xz*-*yz* band states in the BCT structure: the nearly soft intra-band charge fluctuations can also readily lead to pairing. This supports the mechanism suggested by Raghu *et al*. The interband proximity effect will induce a secondary gap of the form Δ_*xy*_(*k*) ≃ *z(k*_*x*_ + *ik*_*y*_) on the 2D xy-band^3^. However, our results show that the charge susceptibility in the *xy*-band is also comparably enhanced, and this suggests a modelling of the SC instability with multiband and mixed-spin character^30^. Whether such microscopics lead to a gap function consistent with that above remains to be seen. However, the Δ(*k*) above, from symmetry considerations, describes a two-fold degenerate spin-triplet state. The response to uniaxial strain discovered by Hicks *et al*.^35^ can possibly be interpreted as - the uniaxial strain will lift this two-fold degeneracy, stabilizing either *p*_*x*_ or *p*_*y*_ symmetry for tensile or compressive strain respectively. In fact, strain must also lead to further anisotropies in the normal state responses, since LDA calculations under strain already show an anisotropy for the xy-(*γ*)-FS sheet under uniaxial strain. It would be interesting to probe this aspect in more detail in future. Moreover, while USC following from quasi-1D xz-yz bands is not of the topological type, the proximity effect restores this aspect^29^. But given the relative weakness of the proximity coupling, one expects that the edge currents using SQUID imaging studies^36^ will have magnitudes much smaller than expected, as apparently seen. Thus, a natural choice for an USC arising first in the quasi-1D bands seems to be qualitatively consistent with constraints mandated by a wide range of data.

However, within the truly multiband view, Scaffidi *et al*.^30^ posit an alternative explanation (high Chern number) for the much smaller edge currents in SQUID data, so the issue is unresolved and requires further study. In common with most other proposals, this idea still does not fully address (*i*) the fact that the true situation is probably somewhere in between the proposals of Rice *et al*.^3^ and Raghu *et al*., and involves all three *d*-bands (see, however^30^), and more importantly, the requirement imposed by spin-orbital entanglement, namely, that the pair symmetry cannot be classified either as spin singlet or triplet when SOC is strong (the renormalized SOC is in fact larger than the renormalized crystal-field splitting in LDA + DMFT studies^7^. In fact, very recent spectroscopic evidence^6^ strongly suggests a complicated k-dependent spin-orbital entanglement. Indeed this spin-orbital entanglement is the source of the intricate momentum dependence of the orbital content seen in different *t*_2*g*_ bands in our GGA results. More evidence obtains from very recent strain data^37^, where increase of T_*c*_ under uniaxial strain is suggested to lead to a transition between two USC states having odd- and even parity: within a scenario of a pair function with an admixture of spin singlet and triplet (due to SO-entanglement), it could be that the relative weight of the singlet-triplet admixture is modified in favor of an even-parity state under strain. These requirements demand a generalization of the argument above to allow all three *d*-bands with an intricate k-dependent orbital and spin structure, resulting in a k-dependent admixture of spin singlet and triplet components into the full USC gap function, and is out of scope of this work.

## Conclusion

To conclude, we have revisited the important issue of detailed characterization of the “normal” state in Sr_2_RuO_4_ using state-of-the-art first-principles correlation (GGA + SOC + DMFT(CTQMC)) approach. The thorough analysis of the single- and two-particle response, transport and NMR allow us to test our results against existing experimental and theoretical results. Our optics data is in good agreement with Katsufuji *et al*.^23^ and Werner *et al*.^25^. We find good agreement with dHvA data from Bergemann *et al*.^4^. INS data agree well with Braden *et al*.^24^,^26^ and NMR and resistivity find good accord with data from Ishida *et al*.^27^ and Mackenzie *et al*.^1^ respectively. Armed with these excellent semiquantitative accord with a wide range of normal state transport and magnetic fluctuation data, we propose that a hitherto unnoticed aspect related to dominant intra-band charge fluctuations from the *xy, xz, yz* bands can potentially emerge as a new “pair glue” for USC in Sr_2_RuO_4_. Assuming that these charge fluctuations are the major contributors to the pair glue, we use symmetry arguments to argue that USC with a required pair symmetry primarily arises on the *α, β*-FS sheets, and induces it on the quasi-2D xy-band via an interband proximity effect. However, in view of the comparable charge susceptibility from the *xy*-band, its contribution will also be comparable to that of the *xz, yz* bands in reality, necessitating a full three-band scenario in an (orbital-dependent) intermediate-to-strong coupling framework. Our study establishes a concrete link between normal state responses and the USC instability, and provides evidence that nearly soft intra-band charge fluctuations in the Hund metal, involving all Ru *d* bands potentially play an important role in fomenting USC in this system.

## Methods

We first perform ab-initio density functional theory calculations within GGA and GGA + SO for Sr_2_RuO_4_ using the full potential linearized augmented plane-wave (FP-LAPW) method as implemented in the WIEN2k code^12^. We perform Wannierization of the Wien2k output bands around the Fermi level via interface programs like WANNIER90^13^, WIEN2WANNIER^14^. This would, in turn, give us the Wannier orbitals around the Fermi level which serve as inputs of the dynamical mean field theory (DMFT) self-consistency calculation. We use hybridization expansion of continuous time quantum Monte-Carlo solver (CT-QMC) extended up to low temperatures (~1 *meV*) in conjunction with DMFT for treating local correlations. We implement maximum entropy method (MEM)^19^ for analytically continuing two-particle dynamic responses to real frequencies.

## Additional Information

**How to cite this article**: Acharya, S. *et al*. First-Principles Correlated Approach to the Normal State of Strontium Ruthenate. *Sci. Rep.* **7**, 43033; doi: 10.1038/srep43033 (2017).

**Publisher's note:** Springer Nature remains neutral with regard to jurisdictional claims in published maps and institutional affiliations.
